# Correlation between immune‐related Tryptophan‐Kynurenine pathway and severity of severe pneumonia and inflammation‐related polyunsaturated fatty acids

**DOI:** 10.1002/iid3.1088

**Published:** 2023-11-20

**Authors:** Baojun Guo, Mingshan Xue, Teng Zhang, Hui Gan, Runpei Lin, Mingtao Liu, Yuhong Liao, Jiali Lyu, Peiyan Zheng, Baoqing Sun

**Affiliations:** ^1^ Department of Clinical Laboratory National Center for Respiratory Medicine, National Clinical Research Center for Respiratory Disease, State Key Laboratory of Respiratory Disease, Guangzhou Institute of Respiratory Health, The First Affiliated Hospital of Guangzhou Medical University (The Key Laboratory of Advanced Interdisciplinary Studies Center, Advanced Interdisciplinary Studies Center) Guangzhou China; ^2^ School of Medicine Henan University Kaifeng Henan China; ^3^ China Institute for Radiation Protection Taiyuan China

**Keywords:** indoleamine 2,3 dioxygenase, kynurenine, N‐3/N‐6 polyunsaturated fatty acids, severe pneumonia

## Abstract

**Background:**

Immune dysfunction and oxidative stress caused by severe pneumonia can lead to multiple organ dysfunction and even death, causing a significant impact on health and the economy. Currently, great progress has been made in the diagnosis and treatment of this disease, but the mortality rate remains high (approximately 50%). Therefore, there is still potential for further exploration of the immune response mechanisms against severe pneumonia.

**Objective:**

This study analyzed the difference in serum metabolic profiles between patients with severe pneumonia and health individuals through metabolomics, aiming to uncover the correlation between the Tryptophan‐Kynurenine pathway and the severity of severe pneumonia, as well as N‐3/N‐6 polyunsaturated fatty acids (PUFAs).

**Methods:**

In this study, 44 patients with severe pneumonia and 37 health controls were selected. According to the changes in the disease symptoms within the 7 days of admission, the patients were divided into aggravation (*n* = 22) and remission (*n* = 22) groups. Targeted metabolomics techniques were performed to quantify serum metabolites and analyze changes between groups.

**Results:**

Metabolomics analysis showed that serum kynurenine and kynurenine/tryptophan (K/T) were significantly increased and tryptophan was significantly decreased in patients with severe pneumonia; HETE and HEPE in lipids increased significantly, while eicosapentaenoic acid (EPA), docosapentaenoic acid (DPA), docosahexaenoic acid (DHA), α‐linolenic acid (linolenic acid, α‐LNA), arachidonic acid (ARA), Dihomo‐γ‐linolenic acid (DGLA), and 13(s)‐hydroperoxylinoleic acid (HPODE) decreased significantly. Additionally, the longitudinal comparison revealed that Linolenic acid, DPA, and Tryptophan increased significantly in the remission group, while and kynurenine and K/T decreased significantly. In the aggravation group, Kynurenine and K/T increased significantly, while ARA, 8(S)‐hydroxyeicosatetraenoic acid (HETE), 11(S)‐HETE, and Tryptophan decreased significantly. The correlation analysis matrix demonstrated that Tryptophan was positively correlated with DGLA, 12(S)‐hydroxyeicosapentaenoic acid (HEPE), ARA, EPA, α‐LNA, DHA, and DPA. Kynurenine was positively correlated with 8(S)‐HETE and negatively correlated with DHA. Additionally, K/T was negatively correlated with DGLA, ARA, EPA, α‐LNA, DHA, and DPA.

**Conclusion:**

This study revealed that during severe pneumonia, the Tryptophan‐Kynurenine pathway was activated and was positively correlated with the disease progression. On the other hand, the activation of the Tryptophan‐Kynurenine pathway was negatively correlated with N‐3/N‐6 PUFAs.

## Graphical abstract

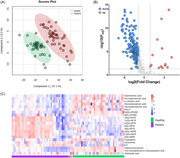

## 1 INTRODUCTION

Currently, the mortality rate associated with severe pneumonia remains high, between 30% and 50%.^1^ The immune system aids the body in combating exogenous pathogens, but the occurrence of acute respiratory distress syndrome and multiple organ failure resulting from an excessive immune response, which triggers an inflammatory storm and oxidative stress, constitutes a significant factor contributing to the high mortality rate associated with severe pneumonia.^2^, ^3^, ^4^

Recent studies have shown that the Tryptophan‐Kynurenine pathway is involved in immune regulation during the inflammatory process.^5^, ^6^, ^7^ Indoleamine 2,3‐dioxygenase (IDO) is a key enzyme in the Tryptophan‐Kynurenine pathway and can be considered a marker of interferon γ (IFN‐γ)‐mediated immune activation, the enzyme's activity can usually be expressed by the ratio of kynurenine to tryptophan (K/T). It plays an important role in inducing immune tolerance by creating a low tryptophan environment and generating metabolites such as kynurenine in local tissues to mediate immunosuppressive effects in the body.^8^

N‐3/N‐6 PUFAs are important indicators of the severity of inflammation. N‐3 PUFAs have anti‐inflammatory effects by reducing the production of inflammatory factors, thereby decreasing the inflammatory response in lung tissue. In contrast, high levels of N‐6 PUFAs can increase the production of inflammatory factors and may worsen the symptoms and severity of pneumonia. Furthermore, the former can attenuate the pro‐inflammatory effects of the latter.^9^, ^10^, ^11^, ^12^

Existing studies have found a negative correlation between the intake of N‐3 PUFAs and IDO activity, which may indicate a reduction in immune activation when the intake of N‐3 PUFAs is higher.^13^ At the same time, N‐3 PUFAs can inhibit LPS‐induced inflammation‐associated depression by suppressing IDO expression.^14^ Furthermore, metabolites of EPA can inhibit the expression of IDO in dendritic cells (DCs) and tumor cells.^15^, ^16^ Nesrine Kamal Bassal et al. have demonstrated that ARA can inhibit the activity of IDO in human monocytes.^17^ This indicates that N‐3/N‐6 PUFAs have a regulatory effect on IDO activity and may influence the development of certain diseases. Therefore, we aim to investigate the changes and correlations between N‐3/N‐6 PUFAs and the Tryptophan‐Kynurenine pathway during severe pneumonia. This research is expected to provide additional theoretical basis for the immune regulatory mechanisms in severe pneumonia.

## 2 METHOD

### 2.1 Patients and control group

A total of 81 subjects were included in the study, including 44 patients with severe pneumonia in the Department of Respiratory Medicine of the First Affiliated Hospital of Guangzhou Medical University, and the remaining 37 health controls. Participants or their families signed informed consent. This study was approved by the Ethics Committee of the First Affiliated Hospital of Guangzhou Medical University.

Inclusion criteria: age ≥18 years; refer to the American Thoracic Society and Infectious Diseases Society of America severe pneumonia standard, meet one of the following major criteria or ≥3 minor criteria. Main criteria: (1) Intubation requires mechanical ventilation; (2) Vascular active drugs are still needed after active fluid resuscitation of septic shock. Secondary criteria: (1) Respiratory frequency ≥30 times/min; (2) Pa02/Fi02 ≤ 250 mmHg; (3) Multilobe infiltration; (4) Disorders of consciousness and/or orientation; (5) Blood urea nitrogen ≥20 mg/dL; (6) Leukopenia (white blood cell [WBC] < 4×10^9^/L); (7) Thrombocytopenia (platelet [PLT] < 100 × 10^9^/L); (8) Hypothermic (central body temperature <36℃); (9) Hypotension requires fluid resuscitation.

Exclusion criteria: age ≤18 years; end‐stage renal disease, end‐stage liver disease, diabetes, active pulmonary tuberculosis, lung transplantation, noninfectious interstitial lung disease, pulmonary embolism, severe immunosuppression, malignant tumor, pregnancy, hemodialysis, blood transfusion, and plasma exchange patients.

Serum samples and clinical data were collected at admission and 1 week after admission. Patients were divided into exacerbation and remission groups based on their condition at the two time points. Evaluation was performed by three clinicians and two researchers based on the patient's examination results or symptoms, including pneumonia severity index (PSI) score, pulmonary imaging lesion range, inflammatory markers, arterial blood gas, and so forth.

### 2.2 Sample collection and preparation

Collection: Each patient at least two times during hospitalization, each time take 5 mL venous blood, all in the morning and overnight eating state, stood for 5 min after centrifugation to collect serum supernatant and stored in −80℃ refrigerator.

Preparation: Preparation of serum samples is divided into extraction and derivatization, serum samples thawed at 4℃ Take A, B two groups of EP tube, group A added 1 ppm Prostaglandin D2 (PGD2)‐d4 5 μL, group B added HETE‐d8 5 μL, take 50 uL serum into group A EP tube, then add 600 μL precooled methanol extraction, point shock 10–15 times fully mixed. The mixture was centrifuged at 13000 rpm for 5 min at 4℃, and the supernatant was transferred to the EP tube of group B for nitrogen blowing for about 30 min to obtain pale yellow crystals. Each 5 μL of 1‐Hydroxybenzotriazole (HOBt), 5‐(Diisopropylamino) Amylamine (DIAAA), and O‐(7‐azabenzotriazol‐1‐yl)‐N,N,N',N'‐tetramethyluronium hexafluorophosphate (HATU) was added in turn, and each addition interval required 30–60 s of oscillation. After that, it was diluted to 50 uL with acetonitrile, centrifuged at 13,000 rpm for 5 min at 4℃, and finally transfer 45 μL into the intubation for detection.

### 2.3 Metabolomics detection

Agilent 1290 Infinity LC system (UHPLC; Santa Clara) were used to separated metabolites, which using an consisting of an autosampler, a thermostatically regulated column compartment, and a binary pump with an Agilent Eclipse XDB‐C18 column (2.1 × 100 mm, 1.8 µm). The column temperature was maintained at 40℃ and the autosampler was set at 4℃. Mobile phase A and B were 0.1% formic acid‐containing water and 0.1% formic acid containing acetonitrile, respectively, and the gradient was set as follows: 0–1 min, 15%–23% B; 1–8 min, 23%–33% B; 8–8.5 min, 33%–35% B; 8.5–15.5 min, 35%–47% B; 15.5–16 min, 47%–50% B; 16–23 min, 50%–85% B; 23–25 min, 85%–95% B; 25–28.9 min, 95% B. The injection volume was 1 µL and the flow rate was 0.3 mL/min.

Mass spectrometry was conducted on an Agilent 6550 UHD accurate mass Q‐TOF/MS system with a dual jet stream electrospray ion source (dual AJS ESI). The instrument was operated in positive full scan mode with the following MS parameters: dry gas temperature, 250℃; dry gas flow, 15 L/min; sheath gas temperature, 300℃; sheath gas flow, 11 L/min; nebulizer pressure, 20 psi; capillary voltage, 5000 V; and nozzle voltage, 500 V. Mass spectra were recorded between 200 and 1000 m/z. Accurate mass measurements were obtained by using a low flow of TOF reference mixture (reference masses: m/z 322.0481, 622.0289, 922.0098), containing internal reference masses at m/z 922.0098 (C18H18F24N3O6P3).

### 2.4 Data analysis

Continuous variables in this study were expressed using the median (interquartile range) and categorical variables were expressed as numbers (frequency). Differences in continuous variables were tested using the Mann‐Whitney‐Wilcoxon rank‐sum test (two groups) or the Kruskal‐Wallis test (three or more groups). Categorical data were compared between different groups using the Chi‐square test (two groups) or Fisher's exact test (three or more groups). Correlation analysis was performed using Spearman's correlation coefficient with R package “corrplot.” The significance level for the test was set to .05. Metabolite analysis was conducted using partial least squares discriminant analysis (PLS‐DA) with R package “mixOmics.” Criteria for screening significantly different metabolites were as follows: (1) log2 fold change (FC) > 0.585 or <−0.585; (2) false discovery rate (FDR)‐corrected *p* value < .05. All statistical analyses in this study were performed using R software (version 4.0.0), IBM SPSS Statistics (version 26.0.0.0), and MetaboAnalyst 5.0 (https://www.metaboanalyst.ca/home.xhtm).

## 3 RESULTS

### 3.1 Characteristics of participants

Several parameters, including procalcitonin (PCT), pro brain natriuretic peptide (PRO‐BNP), and d‐Dimer, were measured in patients with severe pneumonia and health individuals. The results demonstrated that the patients with severe pneumonia exhibited higher values than the normal range. Furthermore, some parameters in the blood, neutrophil% (NEU%), neutrophil (NEU), and monocyte (MONO), were higher in patients with severe pneumonia than in the health control group (all *p* < .01). On the other hand, the values detected for lymphocyte% (LYM%), eosinophil% (EOS%), basophil (BAS%), lymphocyte (LYM), and hematocrit (HCT) parameters were lower than those in the health control group (all *p* < .01) (Table 1).

### 3.2 Metabolic pathway construction and metabolite comparison

**Figure 1:**
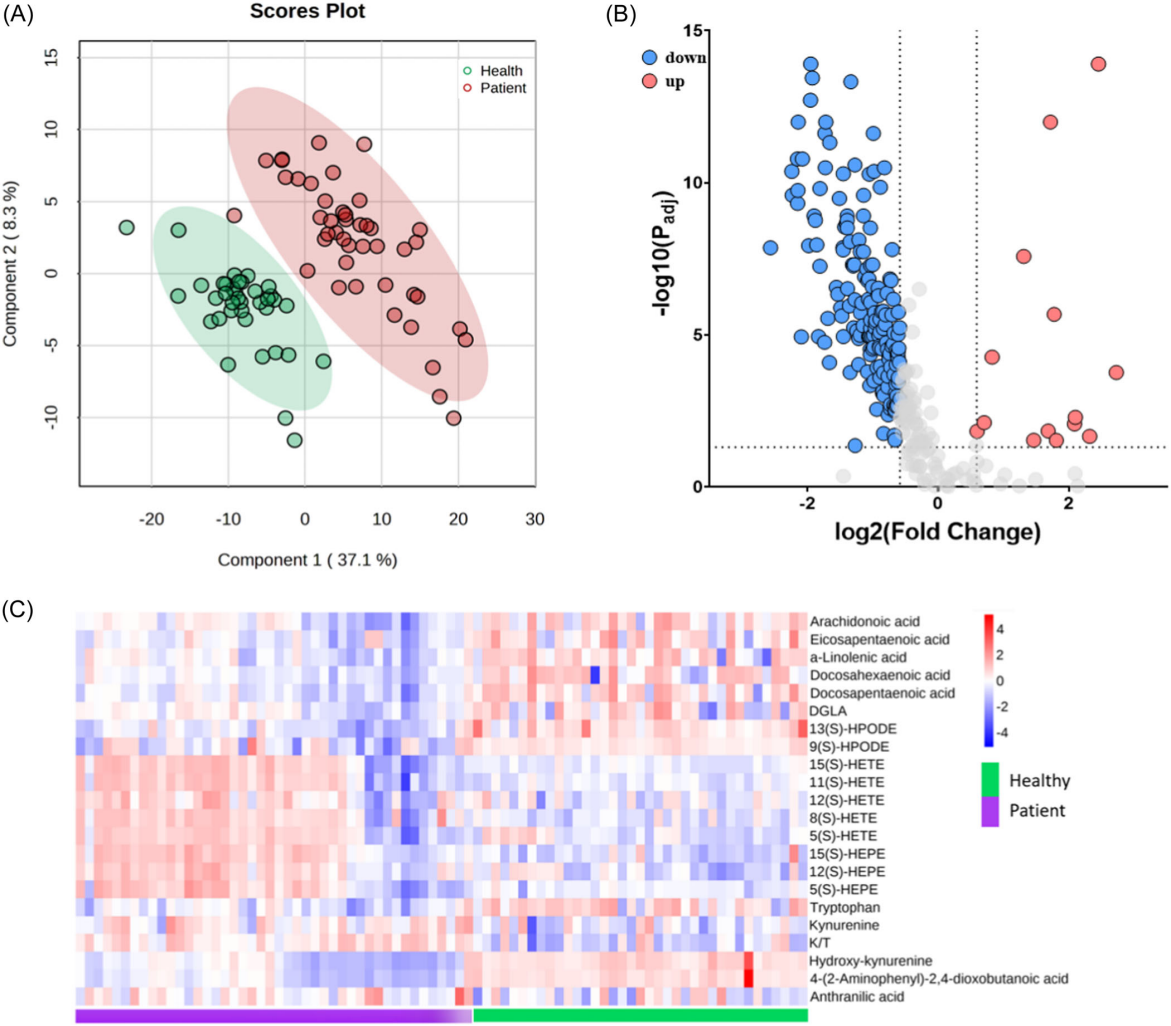
Metabolic analysis of patients with severe pneumonia. (A) Partial least squares discriminant analysis (PLS‐DA) analysis showed a significant separation of serum metabolic phenotypes between patients with severe pneumonia and health people. (B) Volcano plot of targeted metabolomics analysis showed top serum metabolites that increased (red) or decreased (blue) in severe pneumonia compared to controls. (C) Heatmap analysis emphasized the significant changes in N‐3/N‐6 polyunsaturated fatty acids and the kynurenine pathway in patients with severe pneumonia compared with health people.

A targeted liquid chromatography‐tandem mass spectrometry (LC‐MS/MS) analysis identified 272 metabolites. PLS‐DA results showed that the severe pneumonia group was significantly separated from the health control group (Figure 1A). Additionally, good predictive ability and good fitting (R2Y = 0.604, Q2 = 0.572, *p* < .05) were detected by this model. Moreover, a volcano plot performed using serum of patients with severe pneumonia and health individuals revealed that 167 metabolites were downregulated (blue) while 14 metabolites were upregulated (red) compared to the health control group (Figure 1B). We used a heatmap to visualize the Tryptophan‐Kynurenine pathway and the N‐3/N‐6 polyunsaturated fatty acids (Figure 1C).

**Table 1:** Characteristics of included patients and health individuals.

	Patient	Health	*p* Value
N	44.00	37.00	
Sex, female (%)	10.00 (22.70)	8.00 (21.60)	0.906
Age	65.00 (56.75, 72.00)	66.00 (54.00, 75.50)	0.951
PCT, ng/mL	0.18 (0.05, 0.69)	—	—
PRO‐BNP, pg/ml	535.70 (205.80, 2787.00)	—	—
CRP, mg/L	3.34 (1.68, 7.99)	—	—
GLU	6.46 (5.18, 9.71)	—	—
BUN	7.70 (4.83, 16.83)	—	—
Na	139.05 (136.58, 140.88)	—	—
RI	1.11 (0.81, 1.64)	—	—
PSI	121.00 (89.75, 154.00)	—	—
OI	328.50 (227.09, 496.90)	—	—
***Blood cell detection***			
WBC, 10^9/L	9.10 (7.00, 12.40)	6.69 (5.37, 7.61)	<.001
NEU%	81.75 (73.13, 89.43)	59.10 (55.15, 67.45)	<.001
LYM%	8.35 (4.60, 15.83)	27.20 (20.85, 34.00)	<.001
MONO%	7.05 (5.45, 9.40)	7.60 (6.70, 8.90)	.474
EOS%	0.70 (0.10, 2.05)	2.30 (0.90, 4.00)	<.001
BAS%	0.30 (0.20, 0.50)	0.70 (0.40, 0.85)	<.001
NEU, 10^9/L	8.05 (4.85, 10.50)	3.63 (3.17, 4.65)	<.001
LYM, 10^9/L	0.80 (0.53, 1.20)	1.70 (1.36, 2.05)	<.001
MONO, 10^9/L	0.60 (0.40, 1.00)	0.50 (0.40, 0.60)	.007
HCT	0.28 (0.23, 0.35)	0.40 (0.36, 0.43)	<.001
***Coagulation tests***			
PT, second	13.90 (13.40, 15.00)	—	—
INR	1.10 (1.03, 1.19)	—	—
FIB, g/L	4.34 (3.38, 5.83)	—	—
APTT, second	39.60 (35.10, 46.90)	—	—
d‐Dimer, ng/ml	1807.00 (846.00, 3117.00)	—	—
***Blood gas***			
PH	7.41 (7.37, 7.45)	—	—
FiO2	29.00 (21.00, 40.00)	—	—
PaCO2	42.05 (38.78, 47.38)	—	—
PaO2	96.60 (71.18, 114.48)	—	—
SAO2	97.35 (93.85, 98.70)	—	—
PA‐aO2	96.10 (91.05, 136.65)	—	—

**Figure 2:**
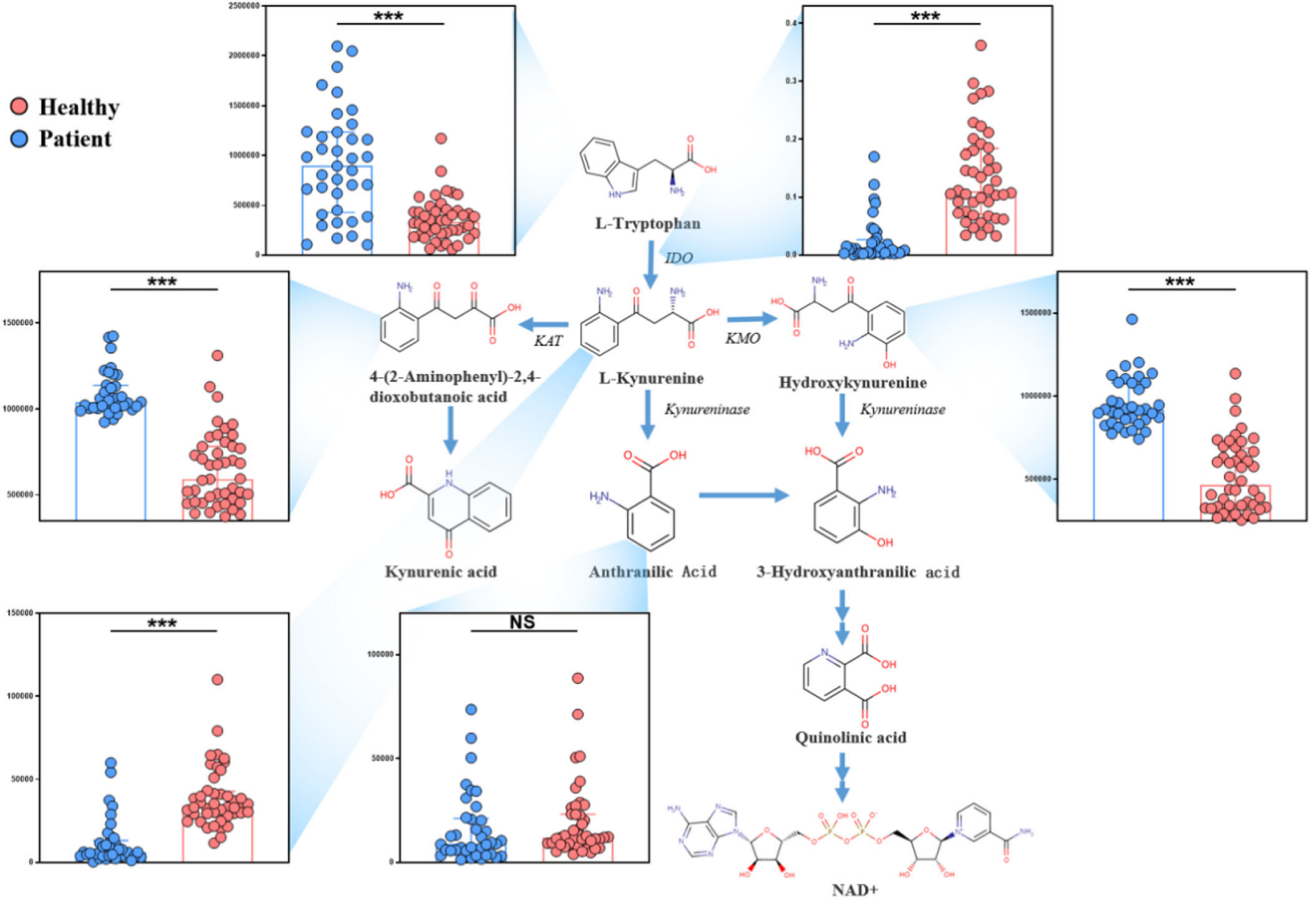
Tryptophan‐Kynurenine metabolic pathway was significantly affected by severe pneumonia, and the asterisk indicated the significance. (*p <.05; **p < .01; ***p < .001).

The Tryptophan‐Kynurenine pathway exhibited significant changes during severe pneumonia (Figure 2)，a significant decrease was detected in the tryptophan, hydroxykynurenine (L‐3‐hydroxykynurenine), and 4‐(2‐Aminophenyl)2,4‐dioxobutanoic acid (AMDA) in the severe pneumonia group than in the health group (Log2 (FC) = −1.36, −0.99, and −0.98, *P*_adj_ = 1.12e‐06, 2.42e‐12, and 4.27e‐11, respectively)， while kynurenine and kynurenine/tryptophan ratio (K/T) increased significantly (Log2 (FC) = 1.71 and 2.45, *P*_adj_ = 1.03e‐12, and 1.25e‐14, respectively). Notably, IDO is a key enzyme in the kynurenine pathway and metabolizes tryptophan to kynurenine. Therefore, in the study, we used the ratio of K/T as a general measure of IDO activity.

**Figure 3:**
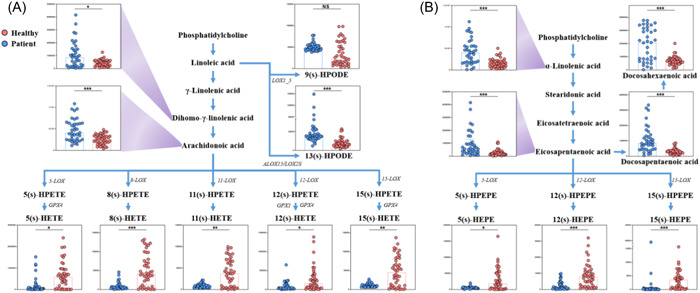
(A) The N‐6 fatty acid metabolic pathway. (B) The N‐3 fatty acid metabolic pathway. The asterisk indicates the significance. (*p < .05; **p < .01; ***p < .001).

Fatty acid metabolism was also significantly affected by severe pneumonia (Figure 3). In this study, N‐6 PUFAs metabolites Dihomo‐γ‐linolenic acid (DGLA), ARA and 13(s)‐hydroperoxylinoleic acid (HPODE) decreased significantly in the severe pneumonia group than in the health group (Log2 (FC) = −1.27, −0.90, and −1.39, *P*_adj_ = 0.04, 2.77e‐05, and 3.26e‐09, respectively), while higher significant levels of the downstream derivatives HETEs were observed (all *P*_adj_ < 0.05). N‐3 fatty acid metabolites EPA, DPA, DHA, and α‐LNA were significantly decreased in the severe pneumonia group than in the health group (Log2 (FC) = −1.74, −1.81, −1.67, and −1.35, *P*_adj_ = 1.78e‐05, 5.58e‐08, 8.35e‐05, and 1.75e‐04, respectively), while 15S‐HEPE, 12S‐HEPE, and 5S‐HEPE increase significantly (Log2 (FC) = 1.30, 1.77, and 2.32, P = 2.72e‐08, 2.16e‐06, and 0.02, respectively).

**Figure 4:**
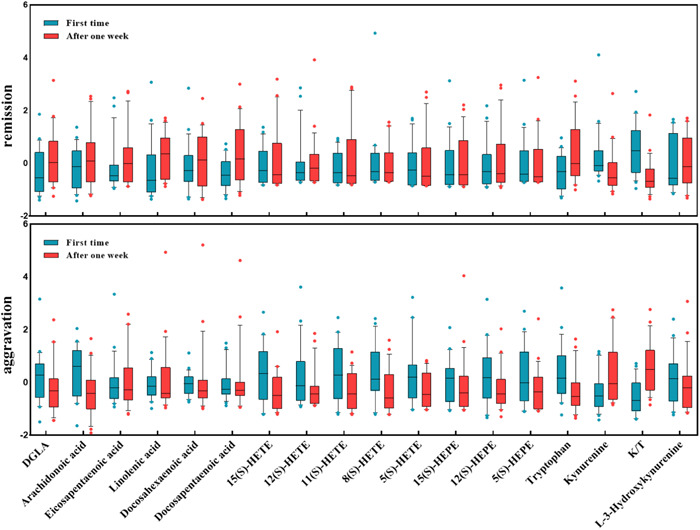
Longitudinal time‐point comparison of severe pneumonia patients at various disease stages.

To perform a longitudinal comparison, the patients with severe pneumonia were divided into two groups: the aggravation group and the remission group, according to the PSI score, arterial blood gas and other indicators during the disease progression. Furthermore, in this experiment, the differences in serum metabolites between the two groups were evaluated at two‐time points, at admission and after 1 week of admission. The results demonstrated that Linolenic acid, DPA, and tryptophan increased significantly in the remission group (*p* = .029, 0.041, and 0.035, respectively), but the kynurenine and K/T decreased significantly in the same group (*p* = .004 and <.001, respectively). On the other hand, the results showed a significant increase in the aggravation group's Kynurenine and K/T (*p* = .039 and <.001, respectively). However, in this group, a significant decrease was detected for ARA, 8(S)‐HETE, 11(S)‐HETE, and tryptophan (*p* = .014, .015, .041, and .021, respectively) (Figure 4). The detailed parameters are reported in Supporting Information Table 1.

**Figure 5:**
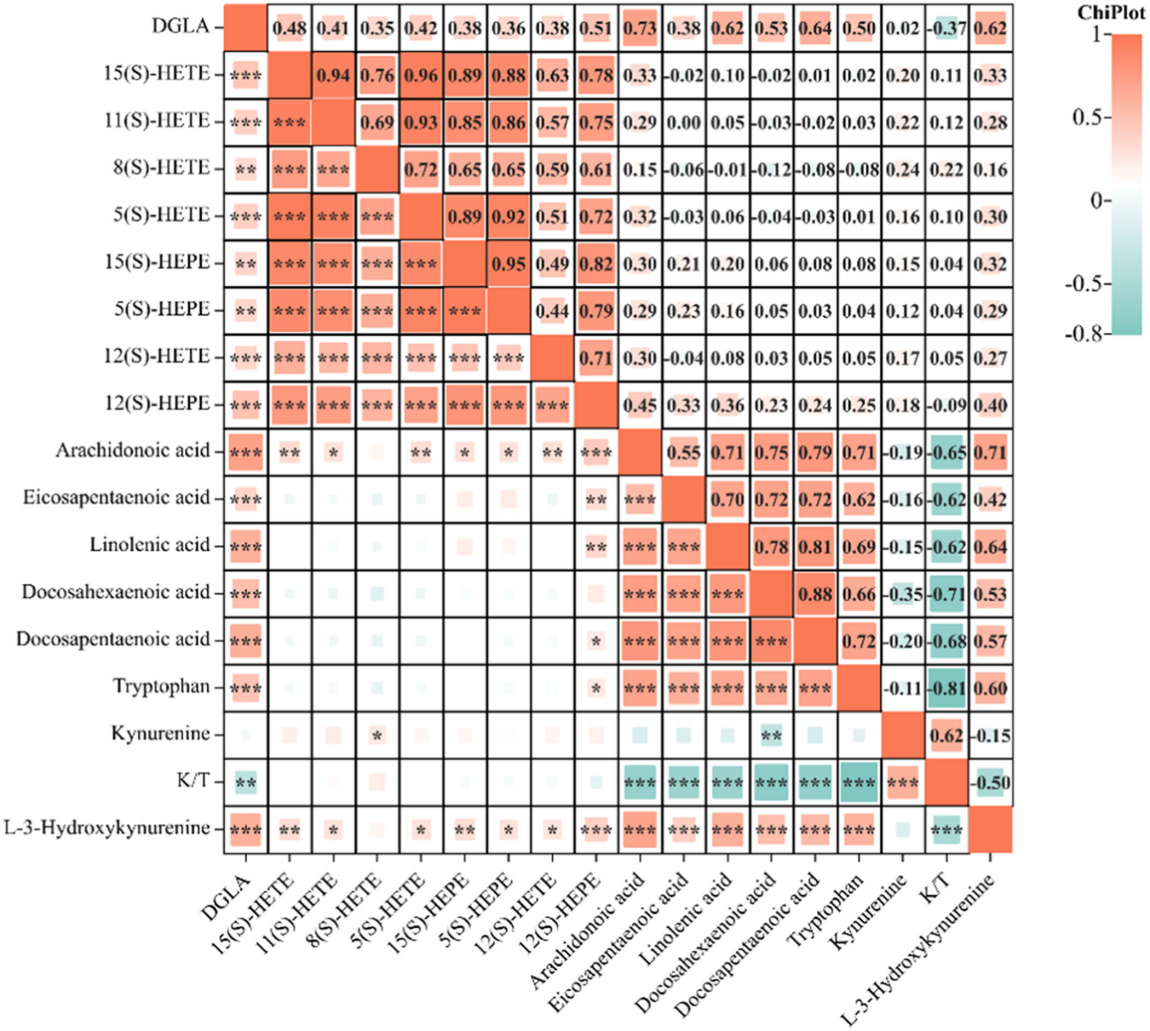
Correlation analysis between the Tryptophan‐Kynurenine pathway indicators and N‐3/N‐6 fatty acid metabolites. The asterisk indicates the significance (*p < .05; **p < .01; ***p < .001).

In this study, the relationships between Tryptophan‐Kynurenine pathway metabolites and N‐3/N‐6 PUFAs metabolites were investigated by Spearman's rank correlation coefficient (Figure 5)，The detailed values of correlation coefficients and P‐values were shown in Supporting Information Table 2. The results demonstrated that Tryptophan was positively correlated with DGLA, 12(S)‐HEPE, ARA, EPA, α‐LNA, DHA, and DPA (*r* = .50, .25, .71, .62, .69, .66, and .72, respectively, *p* < .05), and kynurenine was positively correlated with 8(S)‐HETE (*r* = .24, *p* = .038) and negatively correlated with DHA (r = −.35, *p* < .01). Additionally, K/T was negatively correlated with DGLA, ARA, EPA, α‐LNA, DHA, and DPA (*r* = −.37, −.65, −.62, −.62, −.71, and −.68, respectively, *p* < .01).

## 4 DISCUSSION

Metabolites, derived from human pathophysiological activities, can reflect the state of the body. Therefore, it has been described that metabolomics has great potential for diagnosis and disease prediction. Furthermore, this tool can be applied to explore biochemical mechanisms associated with health conditions. Therefore, targeted metabolomics was used in this study to identify significant alterations in metabolic pathways in patients with severe pneumonia, focusing on the link between the Tryptophan‐Kynurenine pathway, N‐3/N‐6 PUFAs metabolism, and severe pneumonia.

### 4.1 Tryptophan‐Kynurenine pathway regulates the body's immune function

According to the results, the Tryptophan‐Kynurenine pathway was one of the metabolic pathways significantly affected in severe pneumonia. The cross‐sectional study demonstrated that in patients with severe pneumonia, there was a significant decrease in tryptophan, hydroxykynurenine, and AMDA, along with a notable increase in kynurenine and K/T. Furthermore, through longitudinal comparison, it was observed that tryptophan exhibited a significant increase with disease recovery, whereas kynurenine and K/T exhibited a significant decrease. Conversely, with disease progression, kynurenine and K/T showed a significant increase, while tryptophan exhibited a substantial reduction. In addition, it has been described that Tryptophan‐Kynurenine pathway activation could mediate immunosuppressive function.^18^

IDO is a crucial enzyme in the Tryptophan‐Kynurenine pathway, and an innate immune inhibitor produced endogenously by epithelial and dendritic cells.^19^ The enzyme's activity can usually be expressed by the ratio of K/T.^20^ When dendritic cells are stimulated by pro‐inflammatory factors, including IFN‐γ, tumor necrosis factor α, and interleukin‐6, the activity of IDO increases, leading to the accumulation of kynurenine.^21^, ^22^ This, through the aryl hydrocarbon receptor pathway, diminishes the ability of lung epithelial cells to produce immune mediators and suppresses the activity of CD4 + T cells, thereby preventing Th cell‐mediated immune injury induced by acute infections.^23^ On the other hand, the heightened activity of IDO can trigger autophagy in epithelial cells, exerting a negative impact on the secretion of inflammatory cytokines.^24^ In addition, the IDO activity increases can also inhibit T cell proliferation and activation by reducing the energy source.^25^, ^26^

Hydroxykynurenine, 3‐hydroxy‐anthranilic acid, and kynurenic acid are downstream derivatives of kynurenine, and the rate‐limiting enzymes are Kynurenine‐3‐monooxygenase, kynureninase, and kynurenine aminotransferase. It has been described that hydroxykynurenine and 3‐hydroxy‐anthranilic acid are converted from a reduced state to strong oxidants in the presence of molecular oxygen, inducing oxidative stress, injury, and apoptosis of various cells^27^ and inducing lung injury in some inflammatory diseases.^28^ Additionally, AMDA is an intermediate between kynurenine and kynurenic acid, which can be spontaneously converted into kynurenic acid, determining the levels of kynurenic acid in the organism. Therefore, in this study, AMDA was used to evaluate the kynurenic acid levels in patients with severe pneumonia. Furthermore, some research studies have described that kynurenic acid could promote lipid metabolism in adipose tissue, increase cytokine expression of type 2 immune response, and decrease cytokine expression of type 1 immune response.^29^, ^30^, ^31^, ^32^

Therefore, this study hypothesized that the Tryptophan‐Kynurenine pathway is activated in severe pneumonia to prevent self‐damage caused by excessive immune activation. The degree of pathway activation is directly proportional to the severity of the disease.

### 4.2 N‐3/N‐6 polyunsaturated fatty acid metabolism and inflammatory immunity

Changes in lipid metabolism are closely related to inflammatory immunity, energy metabolism, tissue repair, and biosynthesis in pathological conditions. It has been suggested that N‐3 and N‐6 PUFAs have anti‐inflammatory and pro‐inflammatory effects, respectively. N‐3 PUFAs and their metabolites can promote the formation of biologically less active leukotriene B5, and inhibit the cytokines production by inflammatory cells, playing a biological activity in the inflammatory regression period. Therefore, several studies recommended using products containing N‐3 PUFAs in patients with severe pneumonia.^33^, ^34^, ^35^ On the other hand, prostaglandins and leukotrienes can be synthesized by N‐6 PUFAs and promote leukocyte chemotaxis, produce inflammatory factors, and improve vascular permeability. However, some reports demonstrated that an increased intake of N‐6 PUFAs did not impact inflammation.^9^ We hypothesized that the significant decrease of N‐3/N‐6 PUFAs in this study may be due to the large amount of consumption during pathological reactions, which exerts pro‐inflammatory and anti‐inflammatory properties.

Another crucial factor, HETEs, was also evaluated in this study. HETEs, downstream derivatives of arachidonic acid produced by lipoxygenase (LOX) and glutathione peroxidase (GPX) enzymes, are positively correlated with the inflammatory response. This biological pathway can be activated when respiratory epithelial cells are damaged or infected by pathogens. Some subtypes of LOX enzymes can induce inflammation by acting on different sites producing 5(S)‐HETE, 12(S)‐HETE, and 15(S)‐HETE. In addition, these HETEs can accumulate and activate peroxisome proliferator‐activated receptors (PPARs) to enhance the inflammatory response. On the other hand, studies suggested that some HETEs derivatives can stimulate the growth of pulmonary endothelial cells and blood vessels and the occurrence of pulmonary edema.^36^, ^37^, ^38^, ^39^

The substrates of N‐3 PUFAs (such as α‐linolenic acid) and N‐6 PUFAs (including linoleic acid, γ‐linolenic acid, and ARA) are phosphatidylcholines, and it has been described that the activation of these two pathways could inhibit each other.^40^ However, our research findings indicate that levels of N‐3 fatty acid metabolites EPA, DPA, DHA, α‐LNA, and N‐6 fatty acid metabolites ARA were simultaneously reduced in patients with severe pneumonia, while levels of HETEs and HEPEs were elevated. This suggests that under a steady‐state regulatory mechanism, these two pathways exhibit antagonistic and synergistic effects.

### 4.3 Correlation analysis between Tryptophan‐Kynurenine pathway and inflammation‐related polyunsaturated fatty acid metabolism

In this study, we found that severe pneumonia activated the Tryptophan‐Kynurenine pathway and N‐3/N‐6 PUFAs metabolism，while the results obtained from the correlation matrix analysis demonstrated that the Tryptophan‐Kynurenine pathway activation was positively correlated with the metabolism of N‐3/N‐6 PUFAs. Some studies demonstrated that EPA could inhibit IDO expression in tumor cells,^15^ while EPA‐derived metabolite resolvin E1 can induce several biological responses, including inhibition of dendritic cell migration and leukocyte infiltration, modulation of IDO synthesis in dendritic cells, inhibition of T cell activation, and modulation of effector T cell death.^16^ Furthermore, AA, DHA and dihomo‐γ‐linolenic acid are potent inhibitors of IDO in human acute monocytic leukemic THP‐1 cells and primary human monocytes.^17^, ^41^ In addition, some reports revealed that an increase in activity of IDO can promote fatty acid oxidation.^42^ Recently, Ele and collaborators found that IDO can promote the downregulation of essential enzymes related to fatty acid synthesis in CD4 + T cells, inhibit fatty acid synthesis, and CD4 + T cell proliferation and differentiation.^43^ Therefore, we hypothesized that severe pneumonia accelerates the metabolism of N‐3/N‐6 PUFAs and relieves Inhibition of IDO activity, thereby enhancing immunosuppression effect and reducing autoimmune destruction.

However, our study has some limitations. First, it is a small‐scale, single‐center study, and mechanical ventilation and medication interventions in some patients may affect the changes in certain metabolites. Second, the study only demonstrates the correlation between the immune‐related Tryptophan‐Kynurenine pathway, the severity of severe pneumonia, and the inflammation‐related PUFAs, without capturing the interactions between these factors, which would require extensive intervention studies for validation. Lastly, since severe pneumonia is often caused by a mixed infection of multiple pathogens, this study did not differentiate between pathogens. However, it has been found that various bacterial, fungal, and viral infections can induce the expression of IDO,^44^, ^45^, ^46^, ^47^, ^48^, ^49^, ^50^, ^51^, ^52^, ^53^ and tryptophan can serve as a prognostic biomarker for COVID‐19, but future research still needs more detailed classification of the pathogen.^6^, ^54^

## 5 CONCLUSION

In this study, metabolomics analysis was conducted on the serum of patients with severe pneumonia and health individuals. The results showed that the Tryptophan‐Kynurenine pathway was activated in severe pneumonia and positively correlated with disease severity, reducing self‐immune damage and oxidative stress by suppressing immune reactions. In contrast, the severity of the disease was negatively correlated with N‐3/N‐6 PUFAs. Therefore, we conclude that the activation of the Tryptophan‐Kynurenine pathway in patients with severe pneumonia is inversely correlated with N‐3/N‐6 PUFAs. Thus, a relationship between inflammation, the Tryptophan‐Kynurenine pathway, and N‐3/N‐6 PUFAs metabolism was established to evaluate disease severity. This study may also be relevant to the development of precision interventions for the progression and treatment of severe pneumonia. The mechanism by which N‐3/N‐6 PUFAs affect immune regulation in severe pneumonia by regulating the Tryptophan‐Kynurenine pathway is worthy of further exploration.

## AUTHOR CONTRIBUTIONS

*Conceptualization*: Baojun Guo, Mingshan Xue Teng Zhang and Baoqing Sun. *Data curation*: Baojun Guo, Mingshan Xue, Jiali Lyu and Teng Zhang. *Formal analysis*: Baojun Guo Mingshan Xue, Teng Zhang, Runpei Lin, and Mingtao Liu. *Funding acquisition*: Baoqing Sun. *Investigation*: Yuhong Liao, Mingshan Xue, Teng Zhang, Mingtao Liu, Jiali Lyu, and Peiyan Zheng. *Methodology*: Baojun Guo, Teng Zhang, and Hui Gan. *Project administration*: Baoqing Sun and Peiyan Zheng. *Resources*: Runpei Lin, Jiali Lyu, Peiyan Zheng, and Baoqing Sun. *Software*: Baojun Guo, Teng Zhang Runpei Lin, and Hui Gan. *Supervision*: Baojun Guo, Baoqing Sun, and Peiyan Zheng. *Validation*: Mingshan Xue, Yuhong Liao, and Peiyan Zheng. *Visualization*: Baojun Guo, Mingshan Xue, Teng Zhang, and Yuhong Liao. *Writing–original draft*: Baojun Guo, Mingshan Xue, and Teng Zhang. *Writing–review and editing*: Baojun Guo, Mingshan Xue, Teng Zhang, Peiyan Zheng, and Baoqing Sun.

## CONFLICTS OF INTEREST STATEMENT

The authors declare that they have no conflict of interests.

## ETHICS STATEMENT

The study was approved by the Ethics Committee of the First Affiliated Hospital of Guangzhou Medical University (Reference number: GYFYY‐2016‐73).

## Supporting information


**Supplementary Materials**: Supplementary Table 1: Detailed Parameters for Longitudinal Comparison of Disease Changes. Supplementary Table 2: The detailed values of correlation coefficients and *p* values.

Supporting information.

## Data Availability

The datasets generated during and/or analyzed during the current study are not publicly available but are available from the corresponding author on reasonable request. The data that support the findings of this study are available from the corresponding authors.

## References

[1] Martin‐Loeches I , Torres A . New guidelines for severe community‐acquired pneumonia. Curr Opin Pulm Med. 2021;27:210‐215. 33405483 10.1097/MCP.0000000000000760

[2] Uberti F , Ruga S , Farghali M , Galla R , Molinari C . A combination of α‐lipoic acid (ALA) and palmitoylethanolamide (PEA) blocks endotoxin‐induced oxidative stress and cytokine storm: a possible intervention for COVID‐19. J Diet Suppl. 2023;20:133‐155. 34405764 10.1080/19390211.2021.1966152

[3] Ogura S , Shimosawa T . Oxidative stress and organ damages. Curr Hypertens Rep. 2014;16:452. 25011397 10.1007/s11906-014-0452-x

[4] Kim JS , Lee JY , Yang JW , et al. Immunopathogenesis and treatment of cytokine storm in COVID‐19. Theranostics. 2021;11:316‐329. 33391477 10.7150/thno.49713 PMC7681075

[5] Zou MH . Tryptophan‐kynurenine pathway is dysregulated in inflammation, and immune activation. Front Biosci. 2015;20:1116‐1143. 10.2741/4363 PMC4911177 25961549

[6] Michaelis S , Zelzer S , Schnedl WJ , Baranyi A , Meinitzer A , Enko D . Assessment of tryptophan and kynurenine as prognostic markers in patients with SARS‐CoV‐2. Clin Chim Acta. 2022;525:29‐33. 34902346 10.1016/j.cca.2021.12.005 PMC8662911

[7] Haq S , Grondin JA , Khan WI . Tryptophan‐derived serotonin‐kynurenine balance in immune activation and intestinal inflammation. FASEB J. 2021;35:e21888. 34473368 10.1096/fj.202100702R PMC9292703

[8] Wu H , Gong J , Liu Y . Indoleamine 2, 3‐dioxygenase regulation of immune response (review). Mol Med Rep. 2018;17:4867‐4873. 29393500 10.3892/mmr.2018.8537

[9] Innes JK , Calder PC . Omega‐6 fatty acids and inflammation. Prostaglandins, Leukot Essent Fatty Acids. 2018;132:41‐48. 29610056 10.1016/j.plefa.2018.03.004

[10] Ishihara T , Yoshida M , Arita M . Omega‐3 fatty acid‐derived mediators that control inflammation and tissue homeostasis. Int Immunol. 2019;31:559‐567. 30772915 10.1093/intimm/dxz001

[11] Merchant AT , Curhan GC , Rimm EB , Willett WC , Fawzi WW . Intake of n‐6 and n‐3 fatty acids and fish and risk of community‐acquired pneumonia in US men. Am J Clin Nutr. 2005;82:668‐674. 16155282 10.1093/ajcn.82.3.668

[12] Alperovich M , Neuman MI , Willett WC , Curhan GC . Fatty acid intake and the risk of community‐acquired pneumonia in U.S. women. Nutrition. 2007;23:196‐202. 17236748 10.1016/j.nut.2006.11.007 PMC2293281

[13] Berg J , Seyedsadjadi N , Grant R . Saturated fatty acid intake is associated with increased inflammation, conversion of kynurenine to tryptophan, and delta‐9 desaturase activity in healthy humans. Int J Tryptophan Res. 2020;13:117864692098194. 10.1177/1178646920981946 PMC7750901 33414641

[14] Carabelli B , Delattre AM , Waltrick APF , et al. Fish‐oil supplementation decreases Indoleamine‐2,3‐Dioxygenase expression and increases hippocampal serotonin levels in the LPS depression model. Behav Brain Res. 2020;390:112675. 32407816 10.1016/j.bbr.2020.112675

[15] Wang CC , Yang CJ , Wu LH , Lin HC , Wen ZH , Lee CH . Eicosapentaenoic acid reduces indoleamine 2,3‐dioxygenase 1 expression in tumor cells. Int J Med Sci. 2018;15:1296‐1303. 30275755 10.7150/ijms.27326 PMC6158658

[16] Vassiliou EK , Kesler OM , Tadros JH , Ganea D . Bone marrow‐derived dendritic cells generated in the presence of resolvin E1 induce apoptosis of activated CD4+ T cells. J Immunol. 2008;181:4534‐4544. 18802056 10.4049/jimmunol.181.7.4534

[17] Bassal NK , Hughes BP , Costabile M . Arachidonic acid and its COX1/2 metabolites inhibit interferon‐γ mediated induction of indoleamine‐2,3 dioxygenase in THP‐1 cells and human monocytes. Prostaglandins Leukot Essent Fat Acids. 2012;87:119‐126. 10.1016/j.plefa.2012.08.001 22947424

[18] Nakagami Y , Saito H , Katsuki H . 3‐Hydroxykynurenine toxicity on the rat striatum in vivo. Jpn J Pharmacol. 1996;71:183‐186. 8835646 10.1254/jjp.71.183

[19] Mellor AL , Munn DH . IDO expression by dendritic cells: tolerance and tryptophan catabolism. Nat Rev Immunol. 2004;4:762‐774. 15459668 10.1038/nri1457

[20] Guo L , Schurink B , Roos E , et al. Indoleamine 2,3‐dioxygenase (IDO)‐1 and IDO‐2 activity and severe course of COVID‐19. J Pathol. 2022;256:256‐261. 34859884 10.1002/path.5842 PMC8897979

[21] Aldajani WA , Salazar F , Sewell HF , Knox A , Ghaemmaghami AM . Expression and regulation of immune‐modulatory enzyme indoleamine 2,3‐dioxygenase (IDO) by human airway epithelial cells and its effect on T cell activation. Oncotarget. 2016;7:57606‐57617. 27613847 10.18632/oncotarget.11586 PMC5295376

[22] Babcock TA , Carlin JM . Transcriptional activation of indoleamine dioxygenase by interleukin 1 and tumor necrosis factor alpha in interferon‐treated epithelial cells. Cytokine. 2000;12:588‐594. 10843733 10.1006/cyto.1999.0661

[23] Iannitti RG , Carvalho A , Cunha C , et al. Th17/Treg imbalance in murine cystic fibrosis is linked to indoleamine 2,3‐dioxygenase deficiency but corrected by kynurenines. Am J Respir Crit Care Med. 2013;187:609‐620. 23306541 10.1164/rccm.201207-1346OC

[24] Munn DH , Sharma MD , Baban B , et al. GCN2 kinase in T cells mediates proliferative arrest and anergy induction in response to indoleamine 2,3‐dioxygenase. Immunity. 2005;22:633‐642. 15894280 10.1016/j.immuni.2005.03.013

[25] Eleftheriadis T , Pissas G , Yiannaki E , et al. Inhibition of indoleamine 2,3‐dioxygenase in mixed lymphocyte reaction affects glucose influx and enzymes involved in aerobic glycolysis and glutaminolysis in alloreactive T‐cells. Hum Immunol. 2013;74:1501‐1509. 23993986 10.1016/j.humimm.2013.08.268

[26] Metz R , Rust S , Duhadaway JB , et al. IDO inhibits a tryptophan sufficiency signal that stimulates mTOR: a novel IDO effector pathway targeted by D‐1‐methyl‐tryptophan. Oncoimmunology. 2012;1:1460‐1468. 23264892 10.4161/onci.21716 PMC3525601

[27] Dorta E , Aspée A , Pino E , González L , Lissi E , López‐Alarcón C . Controversial alkoxyl and peroxyl radical scavenging activity of the tryptophan metabolite 3‐hydroxy‐anthranilic acid. Biomed Pharmacother. 2017;90:332‐338. 28376401 10.1016/j.biopha.2017.03.082

[28] Mole DJ , McFerran NV , Collett G , et al. Tryptophan catabolites in mesenteric lymph may contribute to pancreatitis‐associated organ failure. Br J Surg. 2008;95:855‐867. 18473343 10.1002/bjs.6112

[29] Agudelo LZ , Ferreira DMS , Cervenka I , et al. Kynurenic acid and Gpr35 regulate adipose tissue energy homeostasis and inflammation. Cell Metab. 2018;27:378‐392. 29414686 10.1016/j.cmet.2018.01.004

[30] Ohshiro H , Tonai‐Kachi H , Ichikawa K . GPR35 is a functional receptor in rat dorsal root ganglion neurons. Biochem Biophys Res Commun. 2008;365:344‐348. 17996730 10.1016/j.bbrc.2007.10.197

[31] Fallarini S , Magliulo L , Paoletti T , de Lalla C , Lombardi G . Expression of functional GPR35 in human iNKT cells. Biochem Biophys Res Commun. 2010;398:420‐425. 20599711 10.1016/j.bbrc.2010.06.091

[32] Wang J , Simonavicius N , Wu X , et al. Kynurenic acid as a ligand for orphan G protein‐coupled receptor GPR35. J Biol Chem. 2006;281:22021‐22028. 16754668 10.1074/jbc.M603503200

[33] Story MJ . Essential sufficiency of zinc, ω‐3 polyunsaturated fatty acids, vitamin D and magnesium for prevention and treatment of COVID‐19, diabetes, cardiovascular diseases, lung diseases and cancer. Biochimie. 2021;187:94‐109. 34082041 10.1016/j.biochi.2021.05.013 PMC8166046

[34] Doaei S , Gholami S , Rastgoo S , et al. The effect of omega‐3 fatty acid supplementation on clinical and biochemical parameters of critically ill patients with COVID‐19: a randomized clinical trial. J Transl Med. 2021;19:128. 33781275 10.1186/s12967-021-02795-5 PMC8006115

[35] Arnardottir H , Pawelzik SC , Öhlund Wistbacka U , et al. Stimulating the resolution of inflammation through omega‐3 polyunsaturated fatty acids in covid‐19: rationale for the covid‐omega‐F trial. Front Physiol. 2021;11:624657. 33505321 10.3389/fphys.2020.624657 PMC7830247

[36] Wang T , Fu X , Chen Q , et al. Arachidonic acid metabolism and kidney inflammation. Int J Mol Sci. 2019;20:3683. 31357612 10.3390/ijms20153683 PMC6695795

[37] Powell WS , Rokach J . Biosynthesis, biological effects, and receptors of hydroxyeicosatetraenoic acids (HETEs) and oxoeicosatetraenoic acids (oxo‐ETEs) derived from arachidonic acid. Biochim et Biophys Acta Mol Cell Biol Lipids. 2015;1851:340‐355. 10.1016/j.bbalip.2014.10.008 PMC5710736 25449650

[38] Brunnström Å , Tryselius Y , Feltenmark S , et al. On the biosynthesis of 15‐HETE and eoxin C4 by human airway epithelial cells. Prostaglandins Other Lipid Mediat. 2015;121:83‐90. 26026713 10.1016/j.prostaglandins.2015.04.010

[39] Pickens CA , Sordillo LM , Zhang C , Fenton JI . Obesity is positively associated with arachidonic acid‐derived 5‐ and 11‐hydroxyeicosatetraenoic acid (HETE). Metabolism. 2017;70:177‐191. 28403941 10.1016/j.metabol.2017.01.034

[40] Nagakura T , Matsuda S , Shichijyo K , Sugimoto H , Hata K . Dietary supplementation with fish oil rich in ω‐3 polyunsaturated fatty acids in children with bronchial asthma. Eur Respir J. 2000;16:861‐865. 11153584 10.1183/09031936.00.16586100

[41] Costabile M , Bassal NK , Gerber JP , Hughes BP . Inhibition of indoleamine 2,3‐dioxygenase activity by fatty acids and prostaglandins: a structure function analysis. Prostaglandins, Leukotrienes Essent Fatty Acids. 2017;122:7‐15. 10.1016/j.plefa.2017.06.010 28735627

[42] Eleftheriadis T , Pissas G , Sounidaki M , et al. Indoleamine 2,3‐dioxygenase, by degrading L‐tryptophan, enhances carnitine palmitoyltransferase I activity and fatty acid oxidation, and exerts fatty acid‐dependent effects in human alloreactive CD4+ T‐cells. Int J Mol Med. 2016;38:1605‐1613. 27667153 10.3892/ijmm.2016.2750

[43] Eleftheriadis T , Pissas G , Antoniadi G , Liakopoulos V , Stefanidis I . Indoleamine 2,3‐dioxygenase depletes tryptophan, activates general control non‐derepressible 2 kinase and down‐regulates key enzymes involved in fatty acid synthesis in primary human CD4+ T cells. Immunology. 2015;146:292‐300. 26147366 10.1111/imm.12502 PMC4582970

[44] Huttunen R , Syrjänen J , Aittoniemi J , et al. High activity of indoleamine 2,3 dioxygenase enzyme predicts disease severity and case fatality in bacteremic patients. Shock. 2010;33:149‐154. 19487973 10.1097/SHK.0b013e3181ad3195

[45] Rajan D , Chinnadurai R , O'Keefe EL , et al. Protective role of indoleamine 2,3 dioxygenase in respiratory syncytial virus associated immune response in airway epithelial cells. Virology. 2017;512:144‐150. 28963880 10.1016/j.virol.2017.09.007 PMC5653408

[46] Jin L , Hu Q , Hu Y , Chen Z , Liao W . Respiratory syncytial virus infection reduces kynurenic acid production and reverses Th17/Treg balance by modulating indoleamine 2,3‐Dioxygenase (IDO) molecules in plasmacytoid dendritic cells. Med Sci Monit. 2020;26:e926763. 33262321 10.12659/MSM.926763 PMC7720431

[47] Murr C , Gerlach D , Widner B , Dierich MP , Fuchs D . Neopterin production and tryptophan degradation in humans infected by *Streptococcus pyogenes* . Med Microbiol Immunol. 2001;189:161‐163. 11388614 10.1007/s430-001-8023-3

[48] Romani L , Puccetti P . Controlling pathogenic inflammation to fungi. Expert Rev Anti Infect Ther. 2007;5:1007‐1017. 18039084 10.1586/14787210.5.6.1007

[49] Romani L , Zelante T , Luca AD , et al. Microbiota control of a tryptophan‐AhR pathway in disease tolerance to fungi. Eur J Immunol. 2014;44:3192‐3200. 25256754 10.1002/eji.201344406

[50] Zelante T , Pieraccini G , Scaringi L , Aversa F , Romani L . Learning from other diseases: protection and pathology in chronic fungal infections. Semin Immunopathol. 2016;38:239‐248. 26382631 10.1007/s00281-015-0523-3

[51] Schmidt SV , Schultze JL . New insights into IDO biology in bacterial and viral infections. Front Immunol. 2014;5:384. 25157255 10.3389/fimmu.2014.00384 PMC4128074

[52] Choera T , Zelante T , Romani L , Keller NP . A multifaceted role of tryptophan metabolism and indoleamine 2,3‐dioxygenase activity in aspergillus fumigatus‐host interactions. Front Immunol. 2018;8:1996. 29403477 10.3389/fimmu.2017.01996 PMC5786828

[53] Suzuki Y , Suda T , Yokomura K , et al. Serum activity of indoleamine 2,3‐dioxygenase predicts prognosis of community‐acquired pneumonia. J Infect. 2011;63:215‐222. 21784100 10.1016/j.jinf.2011.07.003

[54] Thomas T , Stefanoni D , Reisz JA , et al. COVID‐19 infection alters kynurenine and fatty acid metabolism, correlating with IL‐6 levels and renal status. JCI Insight. 2020;5(14). 10.1172/jci.insight.140327 PMC7453907 32559180

